# Multitarget Stool mRNA Test for Detecting Colorectal Cancer Lesions Including Advanced Adenomas

**DOI:** 10.3390/cancers13061228

**Published:** 2021-03-11

**Authors:** Elizabeth Herring, Éric Tremblay, Nathalie McFadden, Shigeru Kanaoka, Jean-François Beaulieu

**Affiliations:** 1 Laboratory of Intestinal Physiopathology, Faculty of Medicine and Health Sciences, Université de Sherbrooke, Sherbrooke, QC J1H 5N4, Canada; elizabeth.herring@usherbrooke.ca (E.H.); eric.tremblay@usherbrooke.ca (É.T.); 2 Centre de Recherche du Centre Hospitalier, Universitaire de Sherbrooke, Sherbrooke, QC J1H 5N4, Canada; nathalie.mc.fadden@usherbrooke.ca; 3 Department of Surgery, Faculty of Medicine and Health Sciences, Université de Sherbrooke, Sherbrooke, QC J1H 5N4, Canada; 4 Department of Gastroenterology, Hamamatsu Medical Center, Naka-ku, Hamamatsu 432-8580, Japan; kanaoka@hmedc.or.jp

**Keywords:** colorectal cancer, advanced adenoma, screening, stool, mRNA

## Abstract

**Simple Summary:**

Colorectal cancer is still one of the deadliest cancers, even though its detection at early stages has been shown to be a key factor for reducing mortality. Screening methods are available, but their efficacy for detecting early-stage lesions is limited. In the present discovery stage study, we used a targeted mRNA assay in the stools to optimize the identification of patients bearing precancerous lesions as well as colorectal cancers at curable stages with only five targets, thus compatible with standard multiplex PCR. Although further validation is required, this assay has high potential for improving colorectal cancer screening efficacy.

**Abstract:**

Current approved non-invasive screening methods for colorectal cancer (CRC) include FIT and DNA-FIT testing, but their efficacy for detecting precancerous lesions that are susceptible to progressing to CRC such as advanced adenomas (AA) remains limited, thus requiring further options to improve the detection of CRC lesions at earlier stages. One of these is host mRNA stool testing. The aims of the present study were to identify specific stool mRNA targets that can predict AA and to investigate their stability under a clinical-like setting. A panel of mRNA targets was tested on stool samples obtained from 102 patients including 78 CRC stage I-III and 24 AA as well as 32 healthy controls. Area under the receiver operating characteristic (ROC) curves were calculated to establish sensitivities and specificities for individual and combined targets. Stability experiments were performed on freshly obtained specimens. Six of the tested targets were found to be specifically increased in the stools of patients with CRC and three in the stools of both AA and CRC patients. After optimization for the choice of the 5 best markers for AA and CRC, ROC curve analysis revealed overall sensitivities of 75% and 89% for AA and CRC, respectively, for a ≥95% specificity, and up to 75% and 95% for AA and CRC, respectively, when combined with the FIT score. Targets were found to be stable in the stools up to 3 days at room temperature. In conclusion, these studies show that the detection of host mRNA in the stools is a valid approach for the screening of colorectal cancerous lesions at all stages and is applicable to a clinical-like setup.

## 1. Introduction

Colorectal cancer (CRC) is one of the few cancer types for which screening has been proven to reduce cancer mortality in average-risk individuals [1]. Indeed, the spread of the disease in terms of local invasion as well as to lymph nodes and distant organs at the time of diagnosis is an important prognostic factor, with five-year survival rates of more than 90% for individuals with localized lesions but only ~10% for those having their CRC metastasized to distal organs [2]. Early detection is thus a key factor in reducing mortality from CRC [3,4]. Advanced adenomas (AA) are also important to detect since they are considered to be the precursors of CRC [5,6], while non-advanced adenomas (<1 cm without advanced histology) may not be associated with increased colorectal cancer risk [6]. Several screening regiments for CRC and AA are recommended such as fecal occult blood testing and colonoscopy. While colonoscopy remains the gold standard for the detection of colorectal lesions, compliance is not optimal owing to discomfort and unpleasant preparation procedures [7]. The risk of complications, cost and access are other limitations of this procedure [8]. On the other hand, the improved immunological version of fecal occult blood testing also referred to as the fecal immunochemical test (FIT), which detects human hemoglobin, has been used for some time with some success [1] but poor precursor lesion detection rates (66–80% sensitivity for CRC but only 10–28% for AA) albeit an excellent specificity (93–95%) limits its effectiveness [3,9,10,11,12]. It is therefore imperative to explore alternate or complementary strategies with the potential to improve CRC screening performance, especially for the detection of cancers at their early stages and AA.

In this context, a number of initiatives have been undertaken over the last ten years, from stool testing as a noninvasive approach [1] to the implementation of personalized CRC screening [13] trying to meet with desirable features for a CRC screening test [3]. Interestingly, many of the stool-based testing strategies are based on the high rate of tumor cell exfoliation into the colon-rectal lumen, a parameter that appears to be independent of blood release [14,15,16,17]. One of the best documented strategies is the FDA-approved multi-target stool DNA test, an approach based on the detection of specific DNA aberrations from the CRC cells shed into the stools in combination with FIT, which results in an improvement of sensitivity for both CRC (92.3%) and AA (42.4%) detection compared to FIT alone, although achieved through a reduction in specificity to 87% thus generating almost three times more false positives [18]. At first sight, the cost–benefit of such new methods for the medical system may temper screening recommendations [19] but the high cost of CRC treatment, particularly for more advanced disease, is considered to improve the cost-effectiveness of CRC screening [20,21]. Furthermore, higher threshold costs for a biomarker test that could significantly increase the sensitivity of AA detection while maintaining reasonable specificity, would likely be cost-effective relative to currently available noninvasive tests [22,23].

Still based on the significant exfoliation of dysplastic cells from colorectal lesions into the lumen, host mRNA has also been investigated in the stools as a potential biomarker. While isolated from purified exfoliated colonocytes [24] or directly extracted from the stools [25,26], host mRNA has been found to be a reliable source of biomarkers for detecting colorectal cancers. Further analysis confirmed the target mRNAs originated from the tumor or surrounding mucosa and that expression was affected by the number of exfoliated tumor cells, exfoliation of inflammatory cells, tumor size and transcript expression level in the tumor but not primary vs. distal location [27]. More recently, based on the analysis of a series of transcripts previously reported to be upregulated in CRC cells [27,28,29] or linked to CRC recurrence [30], it has been demonstrated that the inclusion of a multitarget mRNA assay significantly strengthens both sensitivity and specificity for CRC detection [31,32]. Droplet digital PCR was also evaluated as a potential alternative to qPCR for stool mRNA multiplex analysis [33]. However, one important question that remains to be tested for the validation of a multitarget stool mRNA test pertains to AA detection since, up to now, *ITGA6* is the only target found to be overrepresented in stool samples of patients bearing AA [32]. Another aspect that needs to be evaluated before considering a potential clinical implementation is the robustness of the test under realistic preservation conditions, as mRNA are considered to be relatively susceptible to degradation in the stools [34,35].

## 2. Materials and Methods

### 2.1. Patients and Samples

Two sets of patient samples were used in the study. Both sets were analyzed retrospectively. The first set of samples was collected from patients and healthy controls from the Hamamatsu University School of Medicine with written informed consent. The study was approved by the Institutional Research Ethics Committee of the Hamamatsu University School of Medicine. Complete information about this set has been provided in previous studies [31,32,33] which was further investigated to find the new data reported in this paper. Briefly, the study cohort used herein included 24 patients with AA defined as being 10 mm or larger at their greatest dimension and 78 patients with CRC (24 stage I, 32 stage II and 22 stage III) diagnosed by colonoscopy and histopathology as well as 32 healthy controls. For controls and AA, stool samples were collected before colonoscopy. The FIT was performed on all patients and controls as described [32].

The second set of samples was collected from 3 healthy controls and 3 patients diagnosed with CRC stage II or III by colonoscopy and histopathology from the Centre Hospitalier Universitaire de Sherbrooke (CHUS) with written informed consent. The study was approved by the Institutional Research Ethics Committee of the CHUS. This set of samples was used for mRNA target stability experiments. Each sample was split into 13 aliquots stored under various conditions for up to 5 days as follows: #1, 5 days at −80 °C used as control; #2, 5 days at −20 °C; #3, 5 days at −20 °C with a thaw/freeze cycle; #4–8, 1–5 days at 4 °C and #9–13, 1–5 days at 23 °C.

### 2.2. RNA Isolation, Reverse Transcription, Preamplification, and PCR Amplification

RNA was isolated from fecal samples and reverse transcribed as described previously [27,36]. For preamplification, the TaqMan PreAmp Master Kit (Applied Biosystems, Thermo Fisher Scientific, Mississauga, ON, Canada) was used to provide unbiased, multiplex preamplification of specific amplicons for analysis with TaqMan gene expression assays [33]. Commercially available TaqMan primer and probe mixtures were used for the preamplification of the 27 preselected targets as described before [33] and detailed in Table 1. Quantitative polymerase chain reaction (qPCR) was performed using the TaqMan Gene Expression Assay with conditions described previously [31].

### 2.3. Data Presentation and Statistical Analysis

Stool mRNA data were calculated as copy number per µL of reaction. For each transcript, a standard reference curve was generated using a serial fivefold dilution of a cDNA stock solution of the target sequence quantified on a NanoDrop 1000 Spectrophotometer (NanoDrop, Wilmington, DE, USA). Prism 8 was used for calculating statistics. Comparison mRNA expression (in copy number) in stool controls and patients with AA and CRC stage I-III lesions were expressed as median with interquartile range and analyzed by the Kruskal–Wallis test followed by Dunn’s multiple comparison test. Area under the receiver operating characteristic (ROC) curves were calculated to establish sensitivities and specificities for each marker expressed in % with a 95% confidence interval. Scores were calculated for each marker on a scale of 0 to 3 on the basis of three cut-off values established from the ROC curve: (the lower cut-off corresponding to a sensitivity of 80%, medium cut-off corresponding to a specificity of 90% and higher cut-off corresponding to a specificity of 99%) as established previously [32]. Statistical significance was defined as *p* < 0.05.

## 3. Results

In this study, we first screened 27 specific targets chosen on the basis of their reported over expression in colorectal cancerous lesions. Preliminary evaluation of these using a subset of 30 samples (10 controls, 10 AA and 10 CRC) revealed that 14 were consistently detected in the stools of patients bearing colorectal lesions (Table 1). Further testing with other primer and probe mixtures for poorly detected targets was tried but not further studied herein, since 14 appeared to be enough to run the validation assay considering that for a clinical assay, the multiplex PCR capacity is limited to four to five targets depending on the equipment provided by the manufacturer.

**Figure 1:**
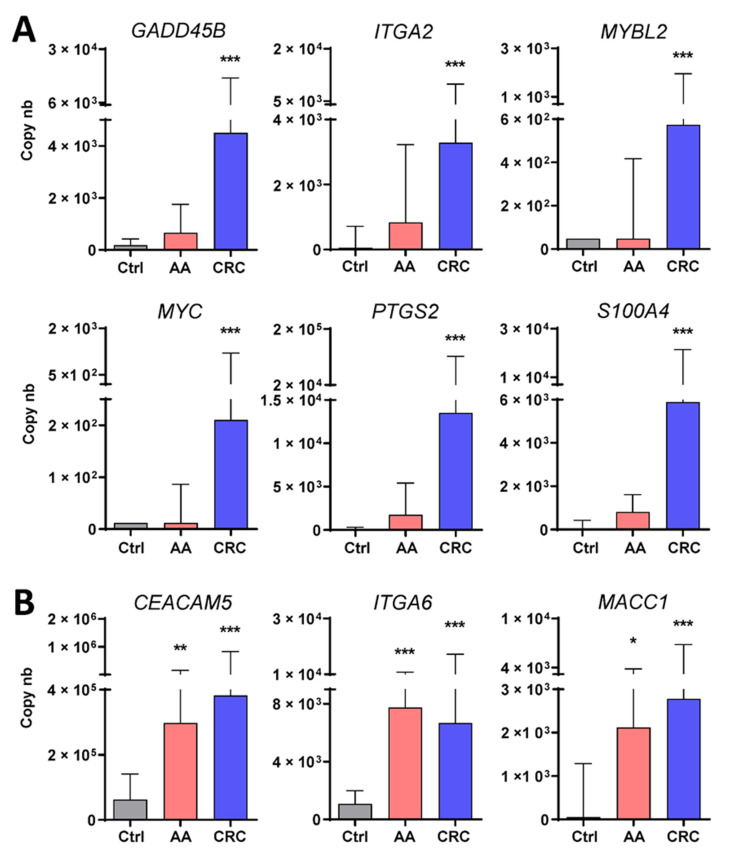
Detection and analysis of selected mRNA targets found to be overrepresented in stool samples of patients with colorectal cancer (CRC) stages I-III (**A**) or advanced adenomas (AA) (**B**). A significant increase was observed for the targets *GADD45B*, *ITGA2*, *MYBL2*, *MYC*, *PTGS2* and *S100A4* in CRC stages I-III as compared to controls (Ctrl) while for three of the targets, *CEACAM5, ITGA6* and *MACC1,* a significant increase was observed in samples from patients with CRC stages I-III or AA as compared to controls (Ctrl). Results are expressed as median (interquartile range) of copy number relative to control patients. * *p* < 0.05, ** *p* < 0.001 and *** *p* < 0.0005 using the Kruskal–Wallis test.

Further investigation of the 14 targets was performed on the set of 132 samples obtained from healthy controls (*n* = 32) and patients bearing colorectal lesions (*n* = 24 AA and 78 CRC). As detailed in Table 1, six of the targets were found to be significantly over-represented in samples from patients with CRC while three identified patients bearing AA or CRC. As shown in Figure 1, the median copy numbers for the transcripts of the first group which included *GADD45B*, *ITGA2*, *MYBL2*, *MYC*, *PTGS2* and *S100A4* were found to be significantly increased in the stools of patients with CRC as compared with the controls, while only three, including *CEACAM5*, *ITGA6* and *MACC1*, were found to be over-represented also in patients with AA.

**Figure 2:**
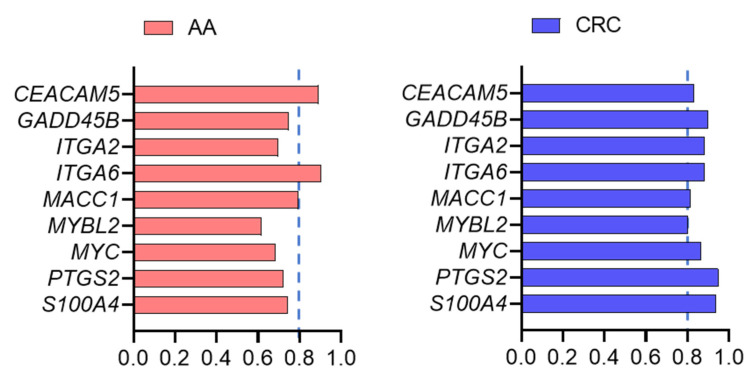
ROC curves were determined for the nine targets characterized in Figure 1 and areas under the curve were calculated to identify those equal or above 0.8 (dotted lines) for AA and CRC.

**Figure 3:**
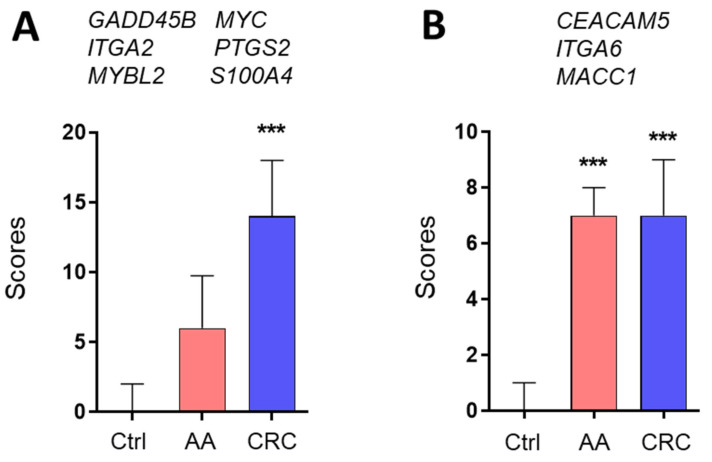
Based on the data from Figure 1 and Figure 2, scores were calculated for the two groups of markers using an algorithm for combining the six targets significant for CRC (**A**) and the three targets significant for AA and CRC (**B**) lesions relative to controls. Results are expressed as median (interquartile range) of scores relative to control patients. *** *p* < 0.0005 using the Kruskal–Wallis test.

ROC curves were calculated for each marker. As expected from the expression levels between control, AA and CRC, the area under the curve (AUC) values were ≥0.8 for all markers for identifying CRC and three markers for identifying AA (Figure 2). The regrouping of the targets was then calculated for the two groups of markers, which can identify CRC only or AA and CRC. As copy numbers varied considerably between the targets, from ~200 for MYC to 40,000 for CEACAM5, individual scores were determined for all targets by attributing a value of 0 to 3 for each patient sample based on the ROC curve cut-off values of the targets, as described in Materials and Methods. Then, an overall score for the each of the two groups of markers was determined for controls and patients with AA or CRC. The overall score for the six markers of the first group significantly recognized the samples from CRC patients vs. those of the controls while the overall scores of the three markers of the second group distinguished the samples from patients bearing CRC or AA from those of the controls (Figure 3).

ROC curves were then produced for the two groups of targets significant for CRC (Figure 3A, Group A) and for AA and CRC (Figure 3B, Group B). As shown in Table 2 (upper two rows) for >95% specificity, group A (Gr. A) displayed 85.2% sensitivity for CRC but only 45% for AA while group B (Gr. B) showed 79% and 75% sensitivity (for 95% specificity) for CRC and AA, respectively.

**Figure 4:**
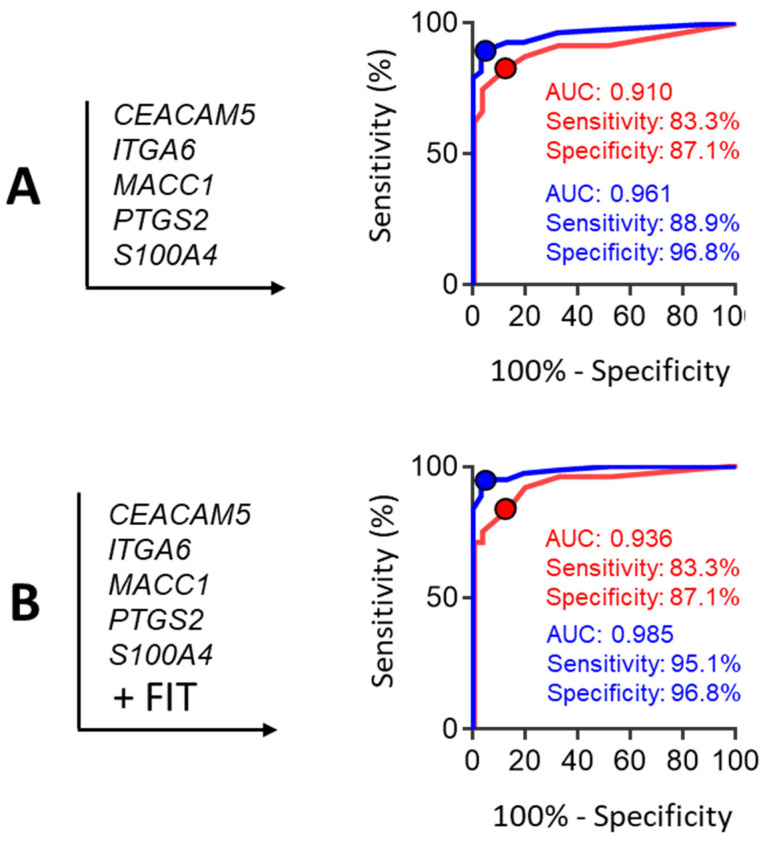
ROC curve analysis of an optimized combination of five of the targets for the detection of patients with AA or CRC as selected from data presented in Table 2. (**A**) ROC curve analysis of the combination of the three targets identified for detecting AA and CRC, CEACAM5, ITGA6 and MACC1 with the two stronger targets for detecting CRC, PTGS2 and S100A4, for AA and CRC. (**B**) Same combination as in A but including the FIT component. AUC is indicated and sensitivity and specificity are provided in % (95% CI).

Considering that the detection 75% of the AA could be achieved using the three markers of the group B (i.e., *CEACAM5*, *ITGA6* and *MACC1*), we then assayed various combinations of markers belonging to the group A in order to improve CRC detection using a maximum of 5 targets while keeping AA detection at 75% (Table 2). The results show that adding the two markers *S100A4* and *PTGS2* significantly improved the rate of CRC detection up to 89% (for 95% specificity) (Table 2, lower row). Corresponding ROC curves are provided in Figure 4A. FIT positivity was 29 % in AA (7/24) and 72% in CRC stage I-III (56/72). Interestingly, considering the result of the FIT in combination with the multi-target score further increased CRC detection up to 95% (for a 97% specificity), but had no significant effect on AA detection (Figure 4B).

**Figure 5:**
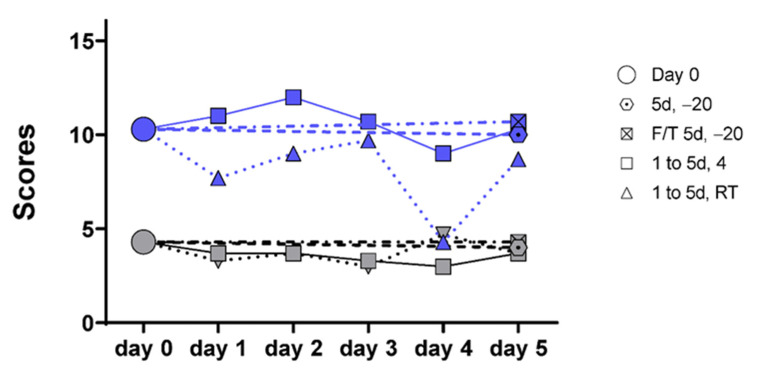
Target stability analyses in stool samples over a 5-day period. Target stability was tested under various conditions of conservation and target detection was monitored throughout the 5 days in samples maintained at −20 °C with (F/T 5d, −20) and without (5d −20) a thaw cycle, at 4 °C (1 to 5d, 4) and at room temperature (1 to 5d, RT). Data for individual targets in copy number are provided in Figure S1. Cumulative scores including the 4 tested targets PTGS2, CEACAM5, ITGA2 and ITGA6 showed that overall, the targets were relatively stable for the five days under all cooled conditions and for three days at room temperature for both controls (Gray symbols) and CRC (Blue symbols).

As the ultimate goal of the study was to evaluate the feasibility of using the multi-target mRNA stool test in a clinical set-up, we evaluated the stability of the mRNA targets in stool samples subjected to various conditions of preservation that mimic the clinical reality. Stool samples were obtained from three controls and three patients diagnosed with CRC. Four of the identified targets in stools were selected for testing, including two for each group identified above: *CEACAM5*, *ITGA6*, *ITGA2*, and *PTGS2*. Conditions to be tested included conventional freezing at −20 °C with and without a thaw cycle, conservation at 4 °C and conservation at room temperature (23 °C), for a 5-day period. As shown in Figure S1, the mRNA targets were found to be relatively stable under all frozen and cooled conditions over the 5-day period while some individual variations were observed in samples maintained at room temperature. Score compilation of the data confirmed the relative stability of the targets for all conditions including ambient temperature for at least 3 days (Figure 5).

## 4. Discussion

In this study, we confirm that a multitarget stool mRNA test represents a powerful assay for detecting patients with colorectal cancers and demonstrate its usefulness to also detect high risk adenomas. One interest of the procedure relies on its relative simplicity considering that high sensitivities and specificities can be obtained with a selection of only five targets, thus compatible with multiplex PCR in stool samples, an approach already in place in the clinic to investigate gastrointestinal infections [37,38].

One strength of the multitarget stool mRNA test presented herein is that transcripts are directly isolated from the stools by conventional extraction methods [25,32] thus being compatible with automation rather than procedures that require enrichment protocols for exfoliated colorectal cells prior to RNA extraction and processing [25,39]. Another strength is the relatively low number of targets required to optimize the assay. It is worth mentioning that an important part of this proof-of-concept study was finding specific targets to identify samples from patients with AA among others that appear to be overrepresented in CRC and then selecting the strongest combination to allow the detection of both AA and CRC. Indeed, some of the targets have been previously assessed for CRC detection [25,32] but it is noteworthy that one of the best markers identified herein for CRC detection, *S100A4*, is reported for the first time. In the same direction, *ITGA6* has been previously reported to be a good candidate for the improvement of AA detection [32] while the current study show that sensitivity and specificity for AA detection can be further improved by combining *ITGA6* with *CEACAM5* and *MACC1*, two targets not yet tested before for this purpose. Further study in this direction would be assisted by the development of specific algorithms where a specific weight is assigned to each marker.

While we agree that our study is preliminary, based on a retrospective analysis of a cohort of patients, as addressed below, it is nevertheless interesting to contextualize the findings that this study, relying on the use of only five mRNA targets, allowed the detection of 75% of the samples obtained from patients with AA and 89% of the samples obtained from patients with CRC, using a specificity of ≥95%. We chose to express the data using this optimal specificity which generates less than 5% of false positives in order to allow a fair comparison to other tests such as FIT as detailed above. Incidentally, integration of the FIT component to the mRNA data increased CRC sensitivity up to 95%, consistent with the fact that the origins of exfoliated cells and blood in the stools are likely to be different [14,15,16,17]. Overall, a multitarget stool mRNA-FIT test allows the detection of 75% of the AA and 95% of the CRC with less than 4% of false positives. These numbers, although from a preliminary study, compared advantageously to any other screening test for colorectal cancerous lesions. As shown with the inclusion of the FIT component, diversification of target types improves sensitivity. In this context, it would be interesting to investigate the potential complementarity of the multitarget stool mRNA test with others approaches also involving stool-derived nucleic acids [18,40,41] or proteins [15,42]. Incidentally, although the extra costs and specific organizational requirements of the multitarget stool mRNA test relative to the FIT are difficult to evaluate at this time, they should be substantially reduced if performed in conjunction with another nucleic acid-based stool test such as the already implemented multi-target stool DNA test [18].

Another finding from this study is the possibility of including a factor for predicting AA vs. CRC, which could provide pertinent information ahead of colonoscopy. Indeed, considered separately, the combination of the three targets *CEACAM5*, *ITGA6* and *MACC1* selected to predict AA provided 75% and 79% sensitivity (for 95% specificity) for AA and CRC, respectively and the two targets *S100A4* and *PTGS2* selected to improve CRC detection provided 29% and 80% sensitivity (for 95% specificity) for AA and CRC prediction, respectively, suggesting that using distinct repertoires of targets for AA and CRC could be used to improve patient stratification for colonoscopy. Specific analysis of *S100A4* and *PTGS2* scores for patients identified as positive in the multi-target stool mRNA test could contribute to discriminating between patients carrying AA vs. those with CRC considering that, for instance, a patient with a score >4.5 for *S100A4* and *PTGS2* displays a 17% probability of having an AA vs. 73% odds of having a CRC.

Finally, the assessment of target stability revealed that stool sample collection to perform the multitarget stool mRNA test does not require specific conditions, being relatively stable for at least 3 days, even at room temperature. Part of this relatively surprising observation may result from the possibility that mRNA degradation is prevented in exfoliated cells, which are the main source of host mRNA in the stools [25,39]. Another part results from the procedure used for selecting the mRNA targets. Incidentally, it was not surprising that only half of the 27 selected targets were amplified in stool samples. The efficient amplification of these targets was also dependent on the use of the TaqMan Gene Expression Assay which was found to be more sensitive and specific than conventional qPCR for stool samples [31] while requiring relatively short intact mRNA sequences.

The limitations of this study, as mentioned above, include the relatively small size of the cohort of patients providing the two sets of samples from only two sites and the fact that the samples were obtained retrospectively. Future investigations should include a larger and multicentric prospective study. However, the relatively low incidence of CRC in the asymptomatic population to be screened complicates this kind of study. Low scale prospective analyses on higher risk cohorts such as FIT positive patients could then be considered.

## 5. Conclusions

In conclusion, this study demonstrates the usefulness of host mRNAs as biomarkers to identify patients carrying curable colorectal cancers as well as precancerous lesions. In the context where various stool-based screening approaches are already implemented or in progress, with their strengths and weaknesses, we suggest that the inclusion of a multitarget stool mRNA component could contribute to getting closer to the “desirable features of a screening test” [3].

## Figures and Tables

**Table 1:** List of specific targets tested.

Gene Name	TaqMan Assay I.D.	Consistently	Over-Represented	
		Detected in Stools	CRC Only	AA and CRC
BGN	Hs00156076_m1			
CEACAM5	Hs00944025_m1	Y		Y
CTNNB1	Hs00355049_m1			
DYNC2H1	Hs00941787_m1			
FAP	Hs00990806_m1			
GADD45B	Hs00169587_m1	Y	Y	
GLI1	Hs00171790_m1			
HMAN1B1	Hs01032463_m1			
HNRNPA2B1	Hs00955384_m1			
INHBA	Hs04187260_m1			
ITGA1	Hs00235006_m1	Y		
ITGA2	Hs01673848_m1	Y		
ITGA6A	Hs01041013_m1	Y	Y	
ITGA6	Hs01041011_m1	Y		Y
KI67	Hs01032434_m1			
KIF3A	Hs01126351_m1			
KIF7	Hs00419527_m1			
MACC1	Hs00766186_m1	Y		Y
MLH1	Hs00179866_m1	Y		
MSH1	Hs00954125_m1	Y		
MTR	Hs01090031_m1			
MYBL2	Hs00942543_m1	Y	Y	
MYC	Hs00153408_m1	Y	Y	
PTGS2	Hs00153133_m1	Y	Y	
S100A4	Hs00243202_m1	Y	Y	
VDAC2	Hs01075603_m1			

All primer and probe mixtures were first tested on a subset of stool samples including controls, AA and CRC to select those that were consistently detectable in the stools. Further analysis on the whole set of samples allowed the selection of those specifically enriched in CRC and AA or only CRC.

**Table 2:** Selection of the best combinations of targets.

		AA						CRC					
		AUC	Sen ^1^	Spe ^1^	YI ^2^	Sen ^3^ Spe ≥95%	Spe ^4^ Sen ≥80%	AUC	Sen ^1^	Spe ^1^	YI ^2^	Sen ^3^ Spe ≥95%	Spe ^4^ Sen ≥80%
Gr. A		0.819	79.1	87.10	0.66	45.30	51.61	0.969	85.19	96.97	0.86	85.19	100.0
Gr. B		0.917	91.67	83.87	0.76	75.00	83.87	0.914	79.01	96.97	0.76	79.01	83.87
Gr. B	+ GADD45B	0.900	75.00	87.88	0.63	70.83	72.73	0.923	79.01	96.97	0.76	79.01	87.88
Gr. B	+ ITGA2	0.900	79.17	90.91	0.70	70.83	66.67	0.929	83.95	96.97	0.81	83.95	96.97
Gr. B	+ MYBL2	0.915	79.17	93.94	0.73	75.00	78.79	0.924	85.19	93.94	0.79	80.25	96.97
Gr. B	+ MYC	0.918	83.33	93.94	0.77	66.67	93.94	0.939	85.19	94.94	0.79	80.25	96.97
Gr. B	+ PTGS2	0.905	79.17	90.32	0.70	66.67	70.97	0.944	86.42	93.55	0.80	81.48	96.97
Gr. B	+ S100A4	0.910	79.17	93.94	0.73	75.00	87.88	0.952	86.42	93.94	0.80	81.48	96.97
Gr. B	+ ITGA2 + S100A4	0.897	79.17	90.91	0.69	70.83	84.85	0.958	86.42	93.94	0.80	82.72	96.97
Gr. B	+ ITGA2 + S100A4	0.890	75.00	93.55	0.69	66.67	80.65	0.952	87.65	93.55	0.81	83.95	100.0
**Gr. B**	**+ PTGS2 + S100A4**	0.910	83.33	87.10	0.70	75.00	87.10	0.961	88.89	96.77	0.86	88.89	96.97

Gr A: GADD45B + ITGA2 + MYBL2 + MYC + PTGS2 + S100A4. Gr B: CEACAM5 + ITGA6 + MACC1. ^1^ Sensitivities (Sen) and Specificities (Spe) were determined based on optimal cut-off values. ^2^ YI: Youden Index. ^3^ Sensitivity for specificity ≥95%. ^4^ Specificity for sensitivity ≥80%. Bold (last row): Best combination.

## Data Availability

The data presented in this study are available on request from the corresponding author.

## Supplementary Materials

The following are available online at https://www.mdpi.com/2072-6694/13/6/1228/s1, Figure S1: Target stability analyses in stool samples over a 5-day period.
