# Supplementary material for: Multitarget Stool mRNA Test for Detecting Colorectal Cancer Lesions Including Advanced Adenomas
Source: Cancers (Basel). 2021 Mar 11;13(6):1228. doi: 10.3390/cancers13061228 (PMC7998137; doi:10.3390/cancers13061228)
Supplement: Supplementary file 1 [file cancers-13-01228-s001.pdf]

cancers

Multitarget Stool mRNA Test for Detecting Colorectal Cancer Lesions Including Advanced Adenomas

Elizabeth Herring, Éric Tremblay, Nathalie McFadden, Shigeru Kanaoka and Jean-François Beaulieu

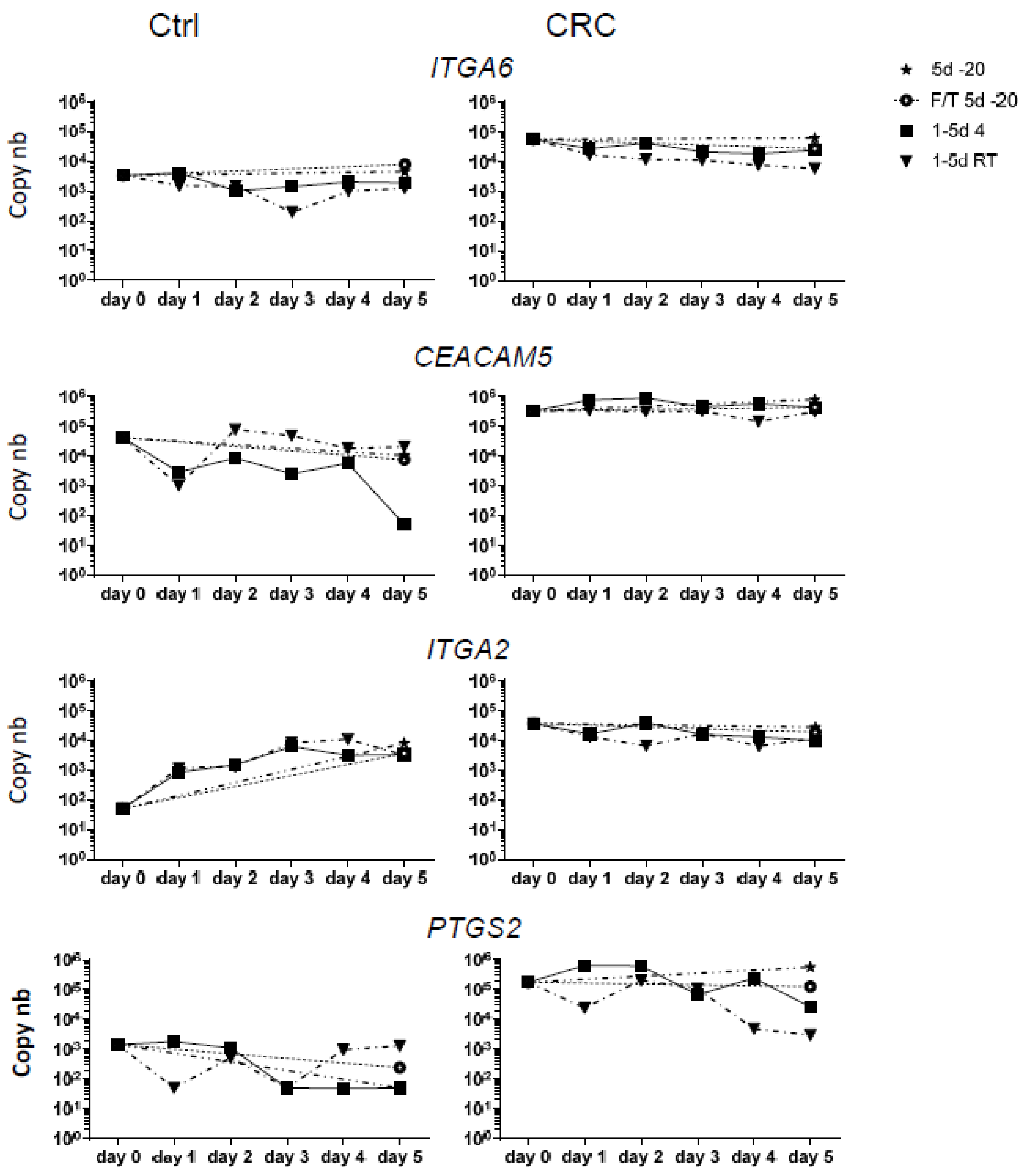

Figure S1. Target stability analyses in stool samples over a 5-day period. Target stability was tested under various conditions of conservation and target detection was monitored throughout the 5 days in samples maintained at −20 °C with (F/T 5d −20) and without (5d −20) a thaw cycle, at 4 °C (1–5d 4) and at room temperature (1-5d RT). As evaluated with ITGA6, CEACAM5, ITGA2 and PTGS2, copy numbers remained relatively stable during the 5 days in both control stool samples (Ctrl) and samples obtained from CRC patients.
